# Synthesis and Cytotoxicity of 2,3-Enopyranosyl C-Linked Conjugates of Genistein

**DOI:** 10.3390/molecules19067072

**Published:** 2014-05-30

**Authors:** Wieslaw Szeja, Grzegorz Grynkiewicz, Tadeusz Bieg, Piotr Swierk, Anna Byczek, Katarzyna Papaj, Radosław Kitel, Aleksandra Rusin

**Affiliations:** 1 Department of Chemistry, Biochemistry and Biotechnology, Silesian Technical University, Krzywoustego 8, 44-100 Gliwice, Poland; E-Mails: tadeusz.bieg@polsl.pl (T.B.); piotr.swierk@polsl.pl (P.S.); anna.byczek@polsl.pl (A.B.); katarzyna.papaj@polsl.pl (K.P.); radoslaw_kitel@o2.pl (R.K.); 2 Pharmaceutical Research Institute, Rydygiera 8, 01-793 Warsaw, Poland; E-Mail: g.grynkiewicz@ifarm.eu; 3 Maria Sklodowska-Curie Memorial Cancer Center & Institute of Oncology, Branch Gliwice, Wybrzeze AK 15, 44-100 Gliwice, Poland

**Keywords:** l-rhamnal, C-glycosylation, genistein conjugates, cell cycle

## Abstract

A series of glycoconjugates, derivatives of genistein containing a C-glycosylated carbohydrate moiety, were synthesized and their anticancer activity was tested *in vitro* in the human cell lines HCT 116 and DU 145. The target compounds **15**–**17** were synthesized by treating ω-bromoalkyl C-glycosides derived from l-rhamnal (**1**) with a tetrabutylammonium salt of genistein. The new, metabolically stable analogs of previously studied O-glycosidic genistein derivatives inhibited proliferation of cancer cell lines through inhibition of the cell cycle.

## 1. Introduction

Natural glycosides have been used directly as biologically active substances (antibiotics, nucleosides, lignans, saponins), but a glycon residue was rarely considered as a functional element in designing new drugs [1,2,3,4,5,6].

2,3-Unsaturated-*O*-glycosides are easily available chiral intermediates in the synthesis of biologically active compounds such as glycopeptide building blocks, oligosaccharides, and modified carbohydrates [7,8,9,10,11]. They have also been employed in the synthesis of some important antibiotics and nucleosides [12,13,14]. Our recent study indicates that introducing unsaturation into the pyranoside ring of flavonoid O-glycosides and glycoconjugates brings about pronounced changes in their biological activity profiles [15,16,17].

**Figure 1:**
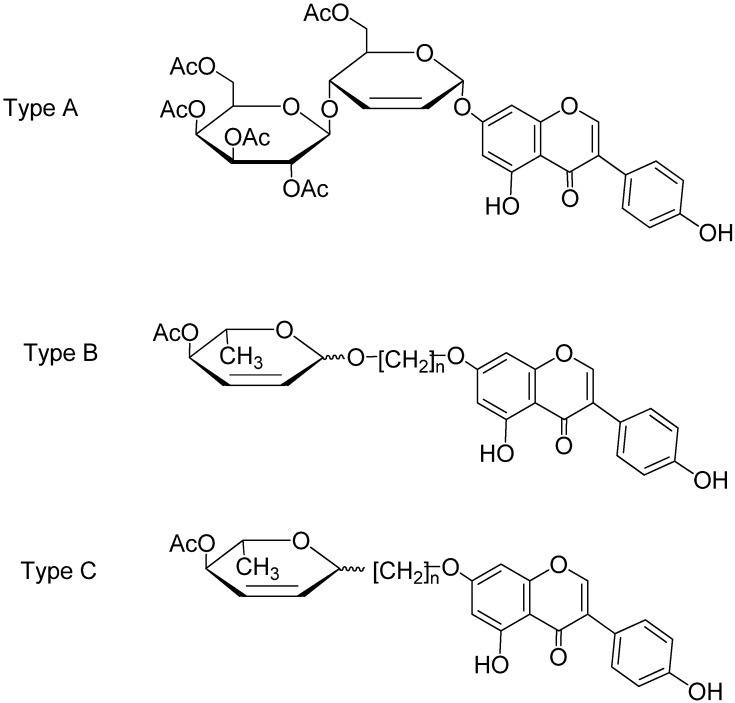
Structure of 2,3-ene-pyranosyl conjugates of genistein.

During our study on the synthetic glycosides of genistein, we have found that some of its 2,3-unsaturated pyranosides (Figure 1, type A) possess a rather unusual and potentially useful ability to interfere with microtubule dynamics and cell cycle progression [17,18,19]. 2,3-Unsaturated pyranosides are, in principle, easily accessible from readily available glycals through Ferrier rearrangements [3,20]. Unfortunately, this well-studied transformation often fails to deliver the required product, particularly in the case of complex and multifunctional aglycons, such as naturally occurring polyalcohols and polyphenols. Thus, genistein for example, does not react with glycals under typical Ferrier rearrangement conditions, even under the influence of new generation Lewis acid catalysts, and the desired unsaturated pyranosides have to be obtained from pre-formed unsaturated anomeric carbonate esters under palladium catalyzed exchange, the reaction which proved nonselective, yielding four distereoisomeric glycoside products [15].

Under these circumstances, structural analogs of unsaturated 7-*O*-genistein pyranosides selected as the first group of derivatives for biological activity study, were the compounds with an aliphatic carbon spacer, which separates a phenolic part and a carbohydrate moiety (Figure 1, type B) [17]. These compounds have retained antiproliferative activity, which prompted the design of further analogs. Since one of the principal problems of glycoside application in medicinal chemistry relates to the question of their integrity in biological media, it appeared that desired modification should lead to replacement of the O-glycosidic by the C-glycosidic bond (Figure 1, type C). Formal removal of the anomeric oxygen atom from aromatic “through spacer” glycosides can be achieved by variety of ways [21]. This paper presents a method for successful C-alkylating transformation of simple glycal, 3,4-di-*O*-acetyl-l-rhamnal (**1**), which is regioselective and stereoselective, and is thus useful for preparation of various chemically and biologically stable analogs of physiologically relevant O-glycosides.

## 2. Results and Discussion

### 2.1. Synthesis of 2,3-Enopyranosyl C-Linked Conjugates of Genistein

The syntheses of C-glycosides have been the subject of intense study [21]. Compounds of this type have been found in Nature and they often possess distinctly different biological activities than their O-glycosidic counterparts [22,23,24,25,26,27,28,29]. As non-hydrolysable analogs of O-glycosides, carbon-linked glycosides have been used as enzyme inhibitors, and they have proved to be involved in important intracellular and intercellular processes [30,31,32,33]. 

In our initial experiments, we have chosen l-rhamnal as a sugar substituent, due to its commercial availability and easy transformation into derivatives of deoxypyranosides—the structural analogs of well-known antibiotic sugars [34,35,36].

We have aimed at synthesis of 2,3-unsaturated C-glycosides (**15**), in which genistein is connected with a sugar segment by an alkyl carbon spacer. The proposed synthetic solution allows the derivation of compounds which can be subsequently transformed into a regular pyranoside (variable derivatization of the double bond C2–C3). Consequently, versatile synthons can be obtained, which are useful for preparation of biomimetic compounds with built-in metabolic stability.

**Scheme 1:**
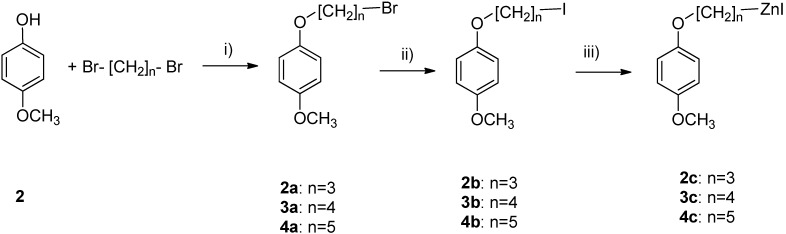
Synthesis of Reformatsky reagents.

Intermediates for the desired C-glycoconjugates (methoxy aryloksyalkylbromides) were prepared by alkylation of phenols with dibromohalides under phase-transfer conditions (PTC) or in the presence of potassium carbonate in acetone solution (Scheme 1) [37,38,39]. The simplicity and efficiency of the PTC technique was demonstrated by McKillop and coworkers in the reaction of phenols with alkyl halides [40]. Treatment of phenols with α,ω-dibromoalkylhalides usually yields diaryloxyalkanes. We have observed that under controlled conditions, only aryloxyalkyl bromides were formed by treatment of phenols with 1,3-dibromopropane, 1,4-dibromobutane, and 1,5-dibromopentane.

Treatment of the bromides with sodium iodide in acetone led to the corresponding aryloxyalkyl iodides [41]. The yield of iodide product was in each case determined with ^1^H-NMR spectroscopy, by measuring the relative intensity of group CH_2_Br towards CH_2_I. The crude product of halogene exchange was used directly for reaction with activated zinc to obtain Reformatsky’s reagent.

*Substrates and conditions*: (**i**) Bu_4_NBr, CH_2_Cl_2_/H_2_O, NaOH, r.t.; (**ii**) NaI, acetone, overnight; (**iii**) Zn.

The reaction of glycals with organozinc reagents has already been a subject of studies presented by Gallagher’s and Orsini’s groups [42,43,44]. They have obtained C-glycosides by applying very reactive organozinc reagents, obtained from esters of ω-haloacids. For the purpose of coupling ω-aryloxy alkyl iodides to 3,4-di-*O*-acetyl-l-rhamnal (**1**) we have worked out a simple and effective method for preparing active zinc dust, using 1,2-dibromoethane in THF at 50 °C, followed by sonification, with addition of chlorotrimethylsilane. 

**Scheme 2:**
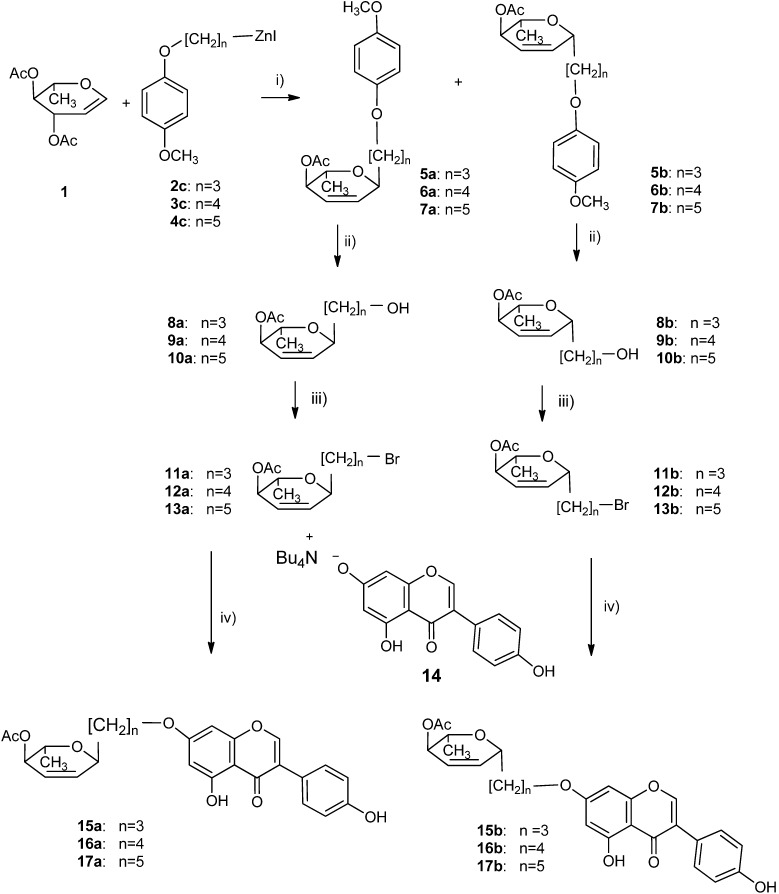
Synthesis of 2,3-enopyranosyl C-linked conjugates of genistein.

Scheme 2 illustrates synthesis of compounds used in biological activity testing. Organozinc reagents were used immediately for coupling with l-rhamnal acetate (**1**) in the presence of BF_3_OEt_2_, which offered better yields then other Lewis acids. Although addition of iodides **2c**–**4c** to 3,4-di-*O*-acetyl-l-rhamnal proceeded with a good yield, the stereoselectivity of the reaction was low, leading to a mixture of α/β C-glycosides (Scheme 2). However, in each case, both stereoisomers could be readily separated by column chromatography. Derivatization of the more acidic phenolic group is easily achieved via its tetra-*n*-butylammonium salt. Thus, by treatment of isoflavone tetra-*n*-butylammonium salt **14** with a near stoichiometric amount of an alkylating agent, 7-*O*-glycosylalkyl derivatives of genistein **15**–**17** can be obtained. The formation of 4'-*O*-derivatives was not observed (^1^H-NMR spectra). The presence of the acetyl group in hex-2,3-enose moiety was intentional and stemmed from positive results obtained in previous study of similar compounds [15]. It is worth noting that, in the final compounds, the acetyl group on the glycone was not removed in the reaction using the tetrabutylammonium salt of genistein. This result is in line with our previous observations [45].

To confirm structures of the products obtained and to identify proton and carbon signals, NMR spectroscopy (^1^H, ^13^C, HSQC and HMBC) was aided by simulation in the MestReNova 8.1.4–12489 program [46]. To determine the stereochemistry of individual C-glycosides we adopted the NOESY technique. In the β isomer, we have observed signal correlation between H-1 and H-5, in contrast to the α isomer, where no correlation between these signals appeared. 

### 2.2. Anticancer Activity in Vitro

The cytotoxicity of six C-linked l-*erythro*-hex-2-enopyranosyl alkyl derivatives of genistein, differing by the length of a linker and the configuration of the C-glycosidic bond (α or β), was tested in HCT 116 (human colorectal cancer cell line) and DU 145 (human prostate cancer cell line). MTT colorimetric assay was used to assess cell viability after 72 h treatment with the tested drugs. In this assay a pale yellow substrate is cleaved by living cells to yield a dark blue formazan product. This process requires active mitochondria, and even freshly dead cells do not cleave significant amounts of MTT.

*Reagents and conditions*: (**i**) BF_3_·EtO_2_, −30 °C, overnight; (**ii**) CAN, 0 °C, 10 min; (**iii**) Ph_3_PBr_2_, r.t., 5 min.; (**iv**) DMF, 50 °C, 8h.

In both tested cell lines, four out of the six l-rhamnal derivatives of genistein demonstrated higher potency than the parent isoflavonoid (Table 1). 

**Table 1:** Cytotoxicity (IC_50_ [µM]) of genistein and its derivatives measured in MTT assay after 72 h treatment of cells with the drugs.

Compound	Cell Line	
	HCT 116	DU 145
Genistein	34.90 ± 9.84	47.29 ± 11.78
**15a**	13.04 ± 2.13	28.02 ± 9.24
**15b**	>25.00 *	>25.00 *
**16a**	2.28 ± 0.62	4.64 ± 2.52
**16b**	35.51 ±17.30	6.85 ± 2.39
**17a**	9.13 ± 6.22	10.09 ± 4.61
**17b**	4.69 ± 3.38	4.90 ± 2.57
Paclitaxel	(3.5 ± 1.1) × 10^−3^	(18.4 ± 3.64) × 10^−3^

* IC_50_ value is higher than the limit of solubility (precipitation of the compound is observed in culture medium).

Compounds **16a** and **17b** showed the greatest antiproliferative activity, with corresponding IC_50_ values lower than 5 µM. Two other compounds, **15a** and **17a**, showed IC_50_ values around 10 µM. The compound **15b** was not active in the concentration up to 25 µM, whereas **16b** was active only in one cell line. Paclitaxel, used as a reference drug, inhibited cell proliferation at low nanomolar concentrations.

Next, we analyzed the influence of the selected glycoconjugates on the cell cycle using a flow cytometer. Univariate analysis of DNA content in cells stained with propidium iodide (PI) allowed us to generate frequency histograms presenting the distribution of cells in three major phases of the cell cycle (G1, S, G2/M), and to detect the apoptotic population, with fractional DNA content (sub-G1), as well as the polyploid population, with DNA content higher than showed by the cells in the G2/M phase. 

**Figure 2:**
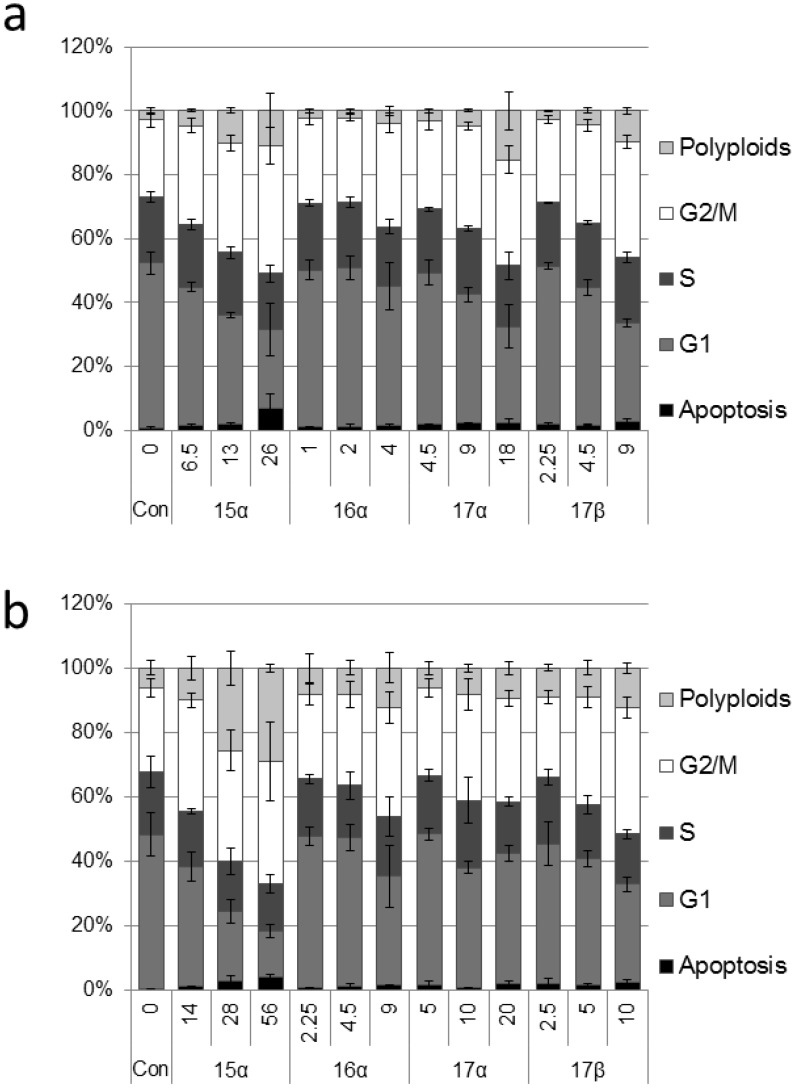
(**a**) Distribution of cell cycle phases in HCT 116 cells treated with the selected genistein derivatives for 24 h. (**b**) Distribution of cell cycle phases in DU 145 cells treated with the selected genistein derivatives for 24 h.

Treatment of both HCT 116 and DU 145 cell lines with compounds **15**–**17** for 24 h altered the cell cycle phase distribution, with a block observed in the G2/M phase (Figure 2). The most profound effect was observed after cell treatment with **15a**. Inhibition of the cell cycle was clearly visible when **15a** was used at a concentration corresponding to ½ of the IC_50_ value, determined in the MTT assay. Compounds **16a**, **17a** and **17b** blocked the cell cycle only when used at the concentration corresponding to 2 × IC_50_ or higher. Worth noting is that all the tested compounds increased the frequency of apoptotic cells in a dose dependent manner. 

Since the method used for determination of the cell cycle phases distribution did not allow us to distinguish whether the cells were stopped in the G2 phase or in mitosis, we decided to calculate the mitotic index using microscope analysis. Compound **15a** induced mitotic arrest, with the mitotic index reaching 9%. The reminder of the tested compounds did not cause mitotic arrest. Microscope examination also allowed us to count the cells with abnormal nuclear morphology. The most common abnormality observed after treatment with the selected drugs was fragmentation of the nucleus. In many cells, multiple large micronuclei were formed, and in extreme cases, the nucleus disintegrated into several micronuclei.

**Figure 3:**
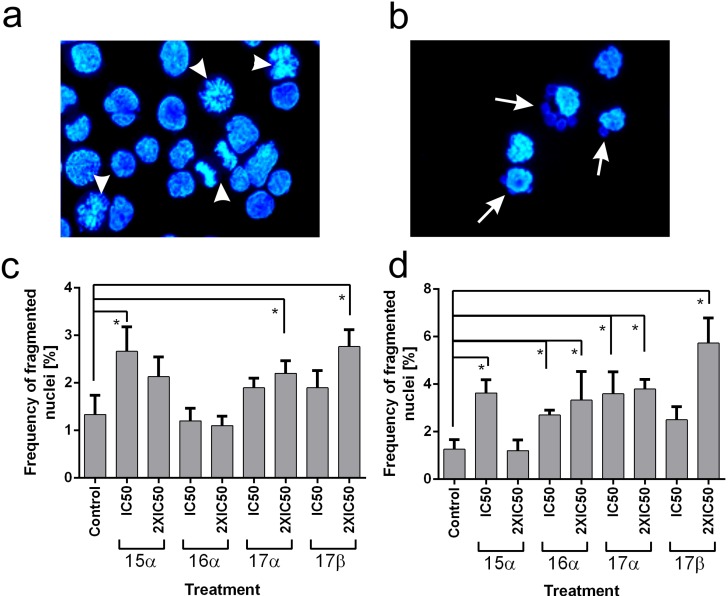
(**a**) Nuclear morphology of control HCT 116 cells. (**b**) Example of abnormal nuclei in HCT 116 cells treated with the compound **17b** for 24 h. (**c**) Frequency of cells with abnormal fragmented nuclei in HCT 116 cell line treated with the tested genistein derivatives for 24 h. (**d**) Frequency of cells with abnormal, fragmented nuclei in DU 145 cell line treated with the tested genistein derivatives for 24 h. Data presented in the (**c**) and (**d**) are mean values obtained in three independent experiments with standard deviations. Statistically important differences between means are marked with asterisks.

These types of nuclear aberrations are characteristic for cells treated with drugs interfering with mitotic spindles [47]. As a result of treatment with either microtubule destabilizing or microtubule stabilizing agents, the chromosomes do not segregate properly during the division into two daughter nuclei. In turn, after chromatin decondensation, the nucleus becomes polyploid or its integrity is impaired and the micronuclei containing a random number of chromosomes are formed. Our observations clearly show that the frequency of cells with fragmented nuclei increased in the tested cell lines after treatment with the derivatives **15a**, **16a**, **17a** and **17b** (Figure 3). 

The number of cells with fragmented nuclei correlated with the polyploidized cells, both indicating, that the tested compounds caused aberrations in cell division. The C-rhamnosides tested in the present study share structural similarities to the derivatives described elsewhere, except for the presence of the C-glycosidic bond instead of the O-glycosidic bond [17]. Presumably, the mechanism of action of these analogs is similar, and is based on inhibiting the cell cycle and interfering with the cell division machinery. 

## 3. Experimental

### 3.1. General Information

Genistein was manufactured at Pharmaceutical Research Institute (Warsaw, Poland), 3,4-di-*O*-Acetyl-l-rhamnal was a commercial reagent (Syntal Chemicals, Gliwice, Poland). The tetra-*n*-butylammonium salt of genistein **7** was prepared as described earlier [48]. The remaining reagents, solvents and sorbents were of commercial origin, certified for research use. Solvents were additionally dried before use as recommended in literature [49,50]. Products of the sugar reactions were recognized based on silica gel TLC RF values (in toluene/ethyl acetate, 2:1). These products were purified by column chromatography performed on silica gel 60 (70–230 mesh, E. Merck, Darmstadt, Germany) with acetone-hexane as the eluent and characterized by HRMS [positive mode, a Mariner PerSeptive Biosystem detector, electrospray + ionization (ESI)], ^1^H-NMR (600 MHz, 300 MHz) and ^13^C-NMR (150 MHz, 75 MHz) spectra, recorded in CDCl_3_ solution (unless state otherwise) with TMS internal standard] on a Varian Inova 600 MHz and 300 MHz apparatus). ^1^H-NMR and ^13^C-NMR signals of some compounds were assigned with the aid of COSY, HSQC and HMBC. Optical rotations were recorded using a JASCO 2000 polarimeter. Melting points were determined on a Koefler apparatus and are not corrected. All evaporations were performed under reduced pressure at 50 °C.

### 3.2. Synthesis of α,ω-Bromoalkyl Ethers of 4-Methoxyphenol

*1-(3-Bromopropoxy)-4-methoxy-benzene* (**2a**): Sodium hydroxide (3.6 g, 1.6 eq.) and 4-methoxyphenol (6.83 g, 55.5 mmol) were dissolved in water (120 mL), then 1,3-dibromopropane (41.6 g, 20.6 mmol) and tetra-N-buthyl ammonium bromide (1.8 g, 5.6 mmol) dissolved in dichloromethane (50 mL) was added. The mixture was stirred for 24 h. The water phase was extracted three times with dichloromethane (100 mL). Combined organic extracts were evaporated, and the crude product was purified by column chromatography (hexane, and hexane: acetone 15:1) to give **2a** as a colorless liquid (12.51 g, 92%). ^1^H-NMR δ (ppm): 2.29 (quintet, 2H, *J* = 6.0 CH_2_CH_2_CH_2_), 3.60 (t, 2H, *J* = 6,3 CH_2_Br), 3.77 (s, 3H, OCH_3_), 4.05 (t, 2H, *J* = 6.0 OCH_2_), 6.84 (s, 4H, H-2, H-3, H-5, H-6). ^13^C-NMR δ (ppm): 30.34 (CH_2_Br), 32.74 (CH_2_CH_2_CH_2_), 55.97 (OCH_3_), 66.29 (OCH_2_), 114.93 (C-3, C-5 or C-2, C-6), 115.81 (C-3, C-5 or C-2, C-6), 153.08 (C-1 or C-4), 154.25 (C-4 or C-1).

*1-(4-Bromobutoxy)-4-methoxy-benzene* (**3a**): Prepared from the 4-methoxyphenol and 1,4-dibromobutane as described before to give **3a** (89%) as a white solid. m.p.: 38–39 °C; ^1^H-NMR (300 MHz, CDCl_3_) δ (ppm): 1.8–1.98 (m, 2H, OCH_2_CH_2_), 1.99–2.12(m, 2H, CH_2_CH_2_Br), 3.48 (t, 2H, *J* = 6.6 Hz, CH_2_Br), 3.76(s, 3H, OCH_3_), 3.94 (t, 2H, *J* = 6.1 Hz, OCH_2_), 6.83 (s, 4H, H-2, H-3, H-5, H-6). ^13^C-NMR (75 MHz, CDCl_3_) δ (ppm): 27.97 (OCH_2_CH_2_), 27.97 (CH_2_CH_2_Br), 33.50 (CH_2_Br), 55.70 (OCH_3_), 67.43(OCH_2_), 114.62(C-3, C-5), 115.37(C-2, C-6), 152.97(C-1), 153.81(C-4).

*1-(5-Bromopentyloxy)-4-methoxy-benzene* (**4a**): Prepared from the 4-methoxyphenol and 1,5-dibromopentane as described before to give **4a** (93%) as a colorless liquid. ^1^H-NMR (300 MHz, CDCl_3_) δ (ppm): 1.55–1.68 (m, 2H, OCH_2_CH_2_CH_2_), 1.72–1.85 (m, 2H, OCH_2_CH_2_), 1.87–2.00 (m, 2H, CH_2_CH_2_Br), 3.43 (t, 2H, *J* = 6.6Hz, CH_2_Br), 3.76 (s, 3H, OCH_3_), 3.92 (t, 2H, *J* = 6.3Hz, OCH_2_), 6.83(s, 4H, H-2, H-3, H-5, H-6). ^13^C-NMR (75 MHz, CDCl_3_) δ (ppm): 24.81(OCH_2_CH_2_CH_2_), 28.51 (OCH_2_CH_2_), 32.48 (CH_2_CH_2_Br), 33.58 (CH_2_Br), 55.70 (OCH_3_), 68.19(OCH_2_), 114.61 (C-3, C-5), 115.40 (C-2, C-6), 153.09 (C-1), 153.75 (C-4). 

### 3.3. Synthesis of α,ω-Iodoalkyl Ethers of 4-Methoxyphenol

*1-(3-Iodopropoxy)-4-methoxybenzene* (**2b**): To a solution of 1-(3-bromopropoxy)-4-methoxybenzene (**2a**, 4 g, 16.32 mmol) in acetone (15 mL) NaI (5.24 g, 34.92 mmol) was added. The reaction mixture was stirred overnight at room temperature, NaBr was filtered off and the filtrate was concentrated. To the residue was added dichloromethane (15 mL), the excess of NaI was filtered off, the filtrate was concentrated to give the crude product (88%) as a yellow oil. ^1^H-NMR δ (ppm): 2.24 (tt, 2H, *J* = 6.0 Hz, CH_2_CH_2_CH_2_), 3.36 (t, 2H, *J* = 6.6, CH_2_I), 3.77 (s, 3H, OCH_3_), 3.98 (t, 2H, *J* = 6.0 OCH_2_), 6.84 (s, 4H, H-2, H-3). ^13^C-NMR δ (ppm): 2.88 (CH_2_I), 33.98 (CH_2_CH_2_CH_2_), 55.97 (OCH_3_), 68.25 (OCH_2_), 114.93 (C-3, C5 or C-2, C-6), 115.83 (C-3, C-5 or C-2, C-6), 153.07 (C-1 or C-4), 154.24 (C-4 or C-1).

*1-(4-Iodobutoxy)-4-methoxybenzene* (**3b**): Prepared from **3a** and 1,4-dibromobutane as described before to give **3b** (91%) as a colorless liquid. ^1^H-NMR (300 MHz, CDCl_3_) δ (ppm): 1.80–2.12 (m, 4H, OCH_2_CH_2_CH_2_CH_2_I); 3.26 (t, 2H, *J* = 6.8 Hz, CH_2_I); 3.77 (s, 3H, OCH_3_); 3.93 (t, 2H, *J* = 6.1 Hz OCH_2_); 6.83 (s, 4H, H-2, H-3, H-5, H-6). ^13^C-NMR (75 MHz, CDCl_3_) δ (ppm): 6.49 (CH_2_I), 30.19 (OCH_2_CH_2_CH_2_CH_2_I), 30.23 (OCH_2_CH_2_CH_2_CH_2_I), 55.72 (OCH_3_), 67.24 (OCH_2_), 114.63 (C-3, C-5), 115.34 (C-2, C-6), 152.98 (C-1), 153.81 (C-4).

*1-(5-Iodopentoxy)-4-methoxybenzene* (**4b**): Prepared from **4a** as described before to give **4b** (91%) as a colorless liquid. ^1^H-NMR (300 MHz, CDCl_3_) δ (ppm): 1.50–1.64 (OCH_2_CH_2_CH_2_CH_2_CH_2_I) 1.71–1.99 (m, 4H, OCH_2_CH_2_CH_2_CH_2_CH_2_I); 3.21(t, 2H, *J* = 6.9Hz, CH_2_I); 3.76(s, 3H, OCH_3_); 3.93(t, 2H, *J* = 6.3Hz OCH_2_); 6.83(s, 4H, H-2, H-3, H-5, H-6). ^13^C-NMR (75 MHz, CDCl_3_) δ (ppm): 6.67 (CH_2_I), 27.12 (OCH_2_CH_2_CH_2_CH_2_CH_2_I), 28.28 (OCH_2_CH_2_CH_2_CH_2_CH_2_I), 33.20 (OCH_2_CH_2_CH_2_CH_2_CH_2_I), 55.70 (OCH_3_), 68.16 (OCH_2_), 114.60 (C-3, C-5), 115.40 (C-2, C-6), 153.08 (C-1), 153.73 (C-4).

### 3.4. General Procedure for the Preparation of Reformatsky Reagents

*1-[3-(4-Methoxyphenoxy)]propyl zinc iodide* (**2c**): A mixture of zinc dust (65 mg, 1 mmol), THF (5 mL), and 1,2-dibromoethane (0.1 mL),was heated at 56 °C for 10 min. After addition of chlorotrimethylsilane (13 μL, 0.15 equiv.) the mixture was sonicated at room temperature for 15 min, then treated with 3-Iodopropyl-4-methoxyphenyl ether (**2b**, 1 mmol, 292 mg) and sonicated for an additional 45 min at room temperature. Next, the mixture was heated to 50 °C and stirred on magnetic stirrer for few hours, cooled to room temperature and concentrated under reduced pressure. The gel-like residue of the reagent was used as such in the unsaturated sugar coupling step.

*1-[4-(4-Methoxyphenoxy)]butyl zinc iodide* (**3c**): Prepared from **3b** as described for 2c. The gel-like residue of the reagent was used as such in the unsaturated sugar coupling step.

*1-[5-(4-methoxyphenoxy)]pentyl zinc iodide* (**4c**): Prepared from **4b** as described for 2c. The gel-like residue of the reagent was used as such in the unsaturated sugar coupling step.

### 3.5. General Procedure for the Synthesis of C-Glycosides

A solution of 3,4-di-*O*-acetyl l-rhamnal (**1**, 200 mg, 0.93 mmol) in dry dichloromethane (2.0 mL) was added to alkylzinc iodide **2c**, **3c**, or **4c** (the gel-like residue, 1 mmol) placed in a 50 mL round bottom flask and the mixture was cooled to −30 °C before addition of boron trifluoride etherate (200 μL, 1.6 mmol) diluted with dichloromethane (1.6 mL). The tightly stoppered reaction flask was left at −30 °C for 24 h and then at 15 °C for another 24 h, after which time the mixture was diluted with dichloromethane (5 mL), transferred to a separation funnel and washed with chilled water. After phase separation the aqueous layer was extracted once with dichloromethane (5 mL) and the combined organic layers were dried with anhydrous sodium sulfate, filtered and evaporated to afford an oily product. The crude product was purified by silica gel column (hexane/acetone 20:1) with TLC control of individual fractions.

*1-C-(4-O-Acetyl-2,3,6-trideoxy-α-L-erythro-hex-2-en-pyranosyl)-3-(4'-methoxyphenoxy) propane* (**5a**): Yellow oil, (181 mg, 60%), 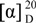 −57.0° (c 1.37, CHCl_3_); ^1^H-NMR (300 MHz, CDCl_3_) δ (ppm): 1.24 (d, *J* = 6.6 Hz, 3H, CH_3_), 1.70–2.00 (m, 4H, CH_2_CH_2_), 2.08 (s, 3H, CH_3_CO), 3.76 (s, 3H, OCH_3_), 3.91 (dq, *J* = 6.6 Hz and 4.9 Hz, 1H, H-5), 3.95 (t, *J*= 6.3 Hz, 2H, OCH_2_), 4.21 (m, 1H, H-1), 4.89 (m, 1H, H-4), 5.78 (ddd, *J*=10.3 Hz, 3.5 Hz and 2.1 Hz, 1H, H-3), 5.92 (ddd, 1H, *J* = 10.4 Hz, 2.2 Hz and 1.3 Hz, 1H, H-2), 6.83 (s, 4H, H-2_ar,_ H-3_ar,_ H-5_ar,_ H-6_ar_). ^13^C-NMR (75 MHz, CDCl_3_) δ (ppm): 17.2 (CH_3_), 21.5 (CH_3_CO), 26.0 (CH_2_CH_2_CH_2_), 30.4 (CH_2_CH_2_CH_2_), 56.0 (OCH_3_), 68.5 (C-5), 68.7 (CH_2_O), 69.9 (C-4), 70.1 (C-1), 114.6 (C-3_ar_, C-5_ar_), 115.7 (C-2_ar_, C-6_ar_), 123.1 (C-3), 134.2 (C-2), 153.4(C-1_ar_), 154.0 (C-4_ar_), 171.0 (C=O). HRMS: calcd for [M+Na]^+^: *m/z* 359.1471. Found: *m/z* 359.1473.

*1-C-(4-O-Acetyl-2,3,6-trideoxy-β-l-erythro-hex-2-en-pyranosyl)-3-(4'-methoxyphenoxy)propane* (**5b**): Yellow oil, (60 mg, 20%), 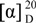 −65.60° (c 1.74, CHCl_3_), ^1^H-NMR (300 MHz, CDCl_3_) δ (ppm): 1.24 (d, *J* = 6.6 Hz, 3H, CH_3_), 1.50–1.90 (m, 6H, CH_2_CH_2_CH_2_), 2.08 (s, 3H, CH_3_CO), 3.76 (s, 3H, OCH_3_), 3.88–3.96 (m, 3H, OCH_2_; H-5), 4.16 (m, 1H, H-1), 4.89 (m, 1H, H-4), 5.76 (ddd, *J* = 10.3 Hz, 3.5 Hz and 2.1 Hz, 1H, H-3), 5.92 (ddd, *J* = 10.4 Hz, 2.1 Hz, and 1.3 Hz, 1H, H-2), 6.83 (s, 4H, H-2ar, H-3_ar,_ H-5_ar_, H-6_ar_). ^13^C-NMR (75 MHz, CDCl_3_) δ (ppm): 17.2 (CH_3_), 21.2 (CH_3_CO), 22.4 (CH_2_CH_2_CH_2_O), 29.2 (CH_2_CH_2_CH_2_O), 33.4 (CH_2_CH_2_CH_2_O), 55.7 (OCH_3_), 68.3 (C-5), 69.6 (C-4), 69.9 (C-1), 114.5 (C-3_ar_, C-5_ar_), 115.4 (C-2_ar_, C-6_ar_), 122.6 (C-3), 134.1 (C-2), 153.1 (C-1_ar_), 153.67 (C-4_ar_), 170.7 (C=O). HRMS: calcd for [M+Na]^+^: *m/z* 359.1471, found: *m/z* 359.1472.

*1-C-(4-O-Acetyl-2,3,6-trideoxy-α-l-erythro-hex-2-enopyranosyl)-4-(4'-methoxyphenoxy)butane* (**6a**): Yellow oil, (186 mg, 60%), 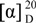 −65.60° (c 1.744, CHCl_3_) ^1^H-NMR (300 MHz, CDCl_3_) δ (ppm): 1.24 (d, 3H, *J* = 6.6 Hz, CH_3_), 1.50–1.90 (m, 6H, CH_2_CH_2_CH_2_), 2.08 (s, 3H, CH_3_CO), 3.76 (s, 3H, OCH_3_), 3.88–3.96 (m, 3H, OCH_2_; H-5), 4.16 (m, 1H, H-1), 4.89 (m, 1H, H-4), 5.76 (ddd, 1H, *J* = 10.3 Hz, *J* = 3.5 Hz, *J* = 2.1Hz, H-3), 5,92 (ddd, 1H, *J* = 10.4 Hz, *J* = 2.1 Hz, *J* = 1.3Hz, H-2), 6.83(s, 4H, H-2_ar,_ H-3_ar_, H-5_ar, _H-6_ar_); ^13^C-NMR (75 MHz, CDCl_3_) δ (ppm): 17.23 (CH_3_), 21.20 (CH_3_CO) 22.36 (CH_2_CH_2_CH_2_CH_2_O), 29.23 (CH_2_CH_2_CH_2_CH_2_O), 33.44 (CH_2_CH_2_CH_2_CH_2_O), 55.68 (OCH_3_), 68.33 (C-5), 68.49 (CH_2_O), 69.65 (C-4), 69.93 (C-1), 114.57 (C-3_ar,_ C-5_ar_), 115.36 (C-2_ar,_ C-6_ar_), 122.57 (C-3), 134.11 (C-2), 153.15 (C-1_ar_), 153.67 (C-4_ar_) 170.74 (C=O).

*1-C-(4-O-Acetyl–2,3,6-trideoxy-β-l-erythro-hex-2-enopyranosyl)-4-(4'-methoxyphenoxy)butane* (**6b**): Yellow oil, (59 mg, 19%), 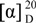 −54.13°, (c 1.84, CHCl_3_), ^1^H-NMR (600 MHz, CDCl_3_) δ (ppm): 1.24 (d, 3H, *J* = 6.0 Hz, CH_3_), 1.50–1.65 (m, 4H, CH_2_CH_2_), 1.71–1.85 (m, 2H, CH_2_CH_2_O) 2.07 (s, 3H, CH_3_CO), 3.57 (dq, 1H, *J* = 8.7 Hz, *J* = 6.2 Hz, H-5), 3.76 (s, 3H, OCH_3_), 3.90 (t, 2H, *J* = 6.5 Hz, OCH_2_), 4.15 (m, 1H, H-1), 5.04 (m, 1H, H-4), 5.70 (ddd, 1H, *J* = 10.3 Hz, *J* = 2.0 Hz, *J* = 2.0 Hz, H-2), 5.80 (ddd, *J* = 10.3, Hz, *J* = 1.5 Hz, *J* = 1.5 Hz, H-3) 6.82 (s, 4H, H-2_ar,_ H-3_ar,_ H-5_ar,_ H-6_ar_). ^13^C-NMR (150 MHz, CDCl_3_) δ (ppm): 18.44 (CH_3_), 21.08 (CH_2_CH_2_CH_2_CH_2_O), 21.35 (CH_3_CO), 29.29 (CH_2_CH_2_CH_2_CH_2_O), 34.96 (CH_2_CH_2_CH_2_CH_2_O), 55.66 (OCH_3_), 68.37 (CH_2_O), 71.27 (C-5), 72.32 (C-4), 74.44 (C-1), 114.56 (C-3_ar_, C-5_ar_), 115.38 (C-2_ar_, C-6_ar_), 125.71 (C-3), 132.92(C-2), 153.17 (C-1_ar_), 153.66 (C-4_ar_) 170.50 (C=O).

*1-C-(4-O-Acetyl-2,3,6-trideoxy-α-l-erythro-hex-2-en-pyranosyl)-5-(4'-methoxyphenoxy) pentane* (**7a**): Yellow oil, (184 mg, 57%), 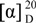 −58.23° (c 2.23, CHCl_3_), ^1^H-NMR (300 MHz, CDCl_3_) δ (ppm): 1.24 (d, 3H, *J* = 6.6 Hz, CH_3_), 1.38–1.71 (m, 6H, CH_2_CH_2_CH_2_CH_2_CH_2_O), 1.78 (q, 2H *J* = 6.6 Hz, CH_2_CH_2_O), 2.08 (s, 3H, CH_3_CO), 3.76 (s, 3H, OCH_3_), 3.84–3.95 (m, 3H, OCH_2_; H-5), 4.14 (m, 1H, H-1), 4.89 (m, 1H, H-4), 5.75 (ddd, 1H, *J* = 10.3 Hz, *J* = 3.4 Hz, *J* = 2.0Hz, H-3), 5.91 (ddd, 1H, *J* = 10.3 Hz, *J* = 2.2 Hz, *J* = 1.2 Hz, H-2), 6.83(s, 4H, H-2_ar,_ H-3_ar,_ H-5_ar,_ H-6_ar_), ^13^C-NMR (75 MHz, CDCl_3_) δ (ppm): 17.23 (CH_3_), 21.21 (CH_3_CO), 25.56 (CH_2_CH_2_CH_2_CH_2_CH_2_O), 25.98 (CH_2_CH_2_CH_2_CH_2_CH_2_O), 29.28 (CH_2_CH_2_CH_2_CH_2_CH_2_O), 33.44 (CH_2_CH_2_CH_2_CH_2_CH_2_O), 55.68 (OCH_3_), 68.40 (C-5; CH_2_O), 69.71 (C-4), 70.06 (C-1), 114.56 (C-3_ar,_ C-5_ar_), 115.36 (C-2_ar,_ C-6_ar_), 122.52 (C-3), 134.19 (C-2), 153.18 (C-1_ar_), 153.64 (C-4_ar_), 170.75 (C=O).

*1-C-(4-O-Acetyl-2,3,6-trideoxy-β-l-erythro-hex-2-en-pyranosyl)-5-(4'-methoxyphenoxy) pentane* (**7b**): Yellow oil, (61 mg, 19%), 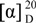 −90.16°, (c 1.96, CHCl_3_), ^1^H-NMR (300 MHz, CDCl_3_) δ (ppm): 1.24 (d, 3H, *J* = 6.0 Hz, CH_3_), 1.35–1.63 (m, 6H, CH_2_CH_2_CH_2_), 1.68–1.84 (m, 2H, CH_2_CH_2_O), 2.08 (s, 3H, CH_3_CO), 3.57 (dq, 1H, *J* = 8.6 Hz, *J* = 6.2 Hz, H-5), 3.76 (s, 3H, OCH_3_), 3.90 (t, 2H, *J* = 6.6 Hz, OCH_2_), 4.14 (m, 1H, H-1), 5.04 (m, 1H, H-4), 5,68 (ddd, 1H, *J* = 10.3 Hz, *J* = 1.9 Hz, *J* = 1.9 Hz, H-2), 5.78 (ddd, *J* = 10.3 Hz, *J* = 1.6 Hz, *J* = 1.6 Hz, H-3) 6,82(s, 4H, H-2_ar,_ H-3_ar,_ H-5_ar,_ H-6_ar_), ^13^C-NMR (75 MHz, CDCl_3_) δ (ppm): 18.47(CH_3_), 21.14 (CH_3_CO), 24.51 (CH_2_CH_2_CH_2_CH_2_CH_2_O), 26.06 (CH_2_CH_2_CH_2_CH_2_CH_2_O), 29.27 (CH_2_CH_2_CH_2_CH_2_CH_2_O), 35.20 (CH_2_CH_2_CH_2_CH_2_CH_2_O) 55.69 (OCH_3_), 68.44 (CH_2_O), 71.31 (C-5), 72.32 (C-4), 74.50 (C-1), 114.56 (C-3_ar,_ C-5_ar_), 115.38 (C-2_ar,_ C-6_ar_), 125.32 (C-3), 132.06 (C-2), 153.20 (C-1_ar_), 153.63 (C-4_ar_), 170.57 (C=O).

### 3.6. General Procedure for Deprotection

A solution of compound **5a** or **5b** (230 mg, 1 mmol) in a mixture of acetonitrile and water (4:1, 8.0 mL) was cooled to 0 °C and treated with ceric ammonium nitrate (CAN, 1.20 g; 2.2 mmol) with stirring. After 10 min a cooling bath was removed and the mixture was allowed to reach ambient temperature. Saturated brine was added (20 mL) and the resulting solution was extracted with ethyl acetate (3 × 20 mL). Combined organic layers were dried with anhydrous sodium sulfate, filtered and concentrated under reduced pressure. The oily residue was purified by column chromatography on silica gel (hexane/acetone 8:1).

*1-C-(4-O-Acetyl-2,3,6-trideoxy-α-l-erythro-hex-2-en-pyranosyl)-3-hydroxypropane* (**8a**): Yellow oil; (82%), 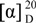 −101.13° (c 1.15, CHCl_3_); ^1^H-NMR (300 MHz, CDCl_3_) δ (ppm): 1.24 (d, *J* = 6.6 Hz, 3H, CH_3_), 1.60–1.80 (m, 4H, CH_2_CH_2_), 2.03 (s, 3H, CH_3_CO), 2.32 (s 1H, OH), 3.66 (m, 2H, CH_2_OH), 3.92 (dq, *J* = 6.5 Hz, 4.5 Hz, 1H, H-5), 4.18 (m,1H, H-1), 4.87 (m, 1H, H-4), 5.79 (ddd, *J* = 10.3 Hz, 3.6 Hz, 2.1 Hz, 1H, H-3), 5.89 (ddd, *J* = 10.3 Hz, 2.1 Hz, 1.2 Hz, 1H, H-2), ^13^C-NMR (75 MHz, CDCl_3_) δ (ppm): 16.76 (CH_3_), 21.2 (CH_3_CO), 29.2 (CH_2_CH_2_CH_2_OH), 30.4 (CH_2_CH_2_CH_2_OH), 62.6 (CH_2_OH), 68.7 (C-5), 69.4 (C-4), 69.9 (C-1), 122.7 (C-3), 134.8 (C-2), 170.7 (C=O). HRMS: calcd for [M+Na]^+^: *m/z* 253.1052, found: 253.1050.

*1-C-(4-O-Acetyl-2,3,6-trideoxy-β-l-erythro-hex-2-en-pyranosyl)-3-hydroxypropane* (**8b**): Yellow oil, (81%), 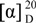 −126.81° (c 0.82, CHCl_3_); ^1^H-NMR (600 MHz, CDCl_3_) δ (ppm): 1.24 (d, *J* = 6.0 Hz, 3H, CH_3_), 1.54–1.63 (m, 1H, CH_2(a)_CH_2_), 1.65–1.75 (m, 3H, CH_2(b)_CH_2_), 2.08 (s, 3H, CH_3_CO), 2.42 (s 1H, OH), 3.60 (dq, 1H, *J* = 8.7 Hz and 6.2 Hz, H-5), 3.64 (m, 2H, CH_2_OH), 4.19 (m, 1H, H-1), 5.05 (m, 1H, H-4), 5.70 (ddd, *J* = 10.3 Hz, 2.1 Hz, 2.1 Hz, 1H, H-2), 5.77 (ddd, *J* = 10.3 Hz, 1.6 Hz, 1.6 Hz, 1H, H-3); ^13^C-NMR (150 MHz, CDCl_3_) δ (ppm): 18.4 (CH_3_), 21.07 (CH_3_CO), 28.3 (CH_2_CH_2_CH_2_OH), 32.0 (CH_2_CH_2_CH_2_OH), 62.6 (CH_2_OH), 71.1 (C-5), 72.43 (C-4), 74.5 (C-1), 125.6 (C-3),132.7 (C-2), 170.5 (C=O). HRMS: calcd for [M+Na]^+^: *m/z* 253.1052, found: *m/z* 253.1056.

*1-C-(4-O-Acetyl-2,3,6-trideoxy-α-l-erytro-hex-2-en-pyranosyl)-4-hydroxybutane* (**9a**): Yellow oil, (83%), 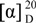 −89.66° (c 1.09, CHCl_3_), ^1^H-NMR (600 MHz, CDCl_3_) δ (ppm): 1.24 (d, 3H, *J* = 6.6 Hz, CH_3_), 1.42–1.72 (m, 6H, CH_2_CH_2_CH_2_), 1.76 (s 1H, OH), 2.08 (s, 3H, CH_3_CO), 3.65 (t, 2H, *J* = 6.5 Hz, CH_2_OH), 3.90 (dq, 1H, *J* = 6.6 Hz, *J* = 4.7 Hz, H-5), 4.14 (m, 1H, H-1), 4.88 (m, 1H, H-4), 5.76 (ddd, 1H *J* = 10.3 Hz, *J* = 3.6 Hz, *J* = 2.1 Hz, H-3), 5.91 (ddd, 1H, *J* = 10.4 Hz, *J* = 2.3 Hz, *J* = 1.3 Hz, H-2). ^13^C-NMR (150 MHz, CDCl_3_) δ (ppm): 16.88 (CH_3_), 21.18 (CH_3_CO), 21.90 (CH_2_CH_2_CH_2_CH_2_OH), 32.48 (CH_2_CH_2_CH_2_CH_2_OH), 33.35 (CH_2_CH_2_CH_2_CH_2_OH), 62.62 (CH_2_OH), 68.52 (C-5), 69.62 (C-4), 69.92 (C-1), 122.50 (C-3), 134.10 (C-2), 170.77 (C=O).

*1-C-(4-O-Acetyl-2,3,6-trideoxy-β-l-erytro-hex-2-en-pyranosyl)-4-hydroxybutane* (**9b**): Yellow oil, (80%), ^1^H-NMR (600 MHz, CDCl_3_) δ (ppm): 1.24 (d, 3H, *J* = 6.1 Hz, CH_3_), 1.42–1.66 (m, 6H, CH_2_CH_2_CH_2_), 1.77 (s 1H, OH), 2.08 (s, 3H, CH_3_CO), 3.57 (dq, 1H, *J* = 8.7 Hz, *J* = 6.2 Hz, H-5), 3,65(t, 2H, *J* = 6.5 Hz, CH_2_OH), 4.15 (m, 1H, H-1), 5.03(m, 1H, H-4), 5.68 (ddd, 1H, *J* = 10.3 Hz, *J* = 2.2 Hz, *J* = 2.2 Hz, H-2), 5.78 (ddd, 1H, *J* = 10.3 Hz, *J* = 1.7 Hz, *J* = 1.7Hz, H-3),^13^C-NMR (150 MHz, CDCl_3_) δ (ppm): 18.44 (CH_3_), 20.94 (CH_3_CO), 21.12 (CH_2_CH_2_CH_2_CH_2_OH), 32.56 (CH_2_CH_2_CH_2_CH_2_OH), 34.87 (CH_2_CH_2_CH_2_CH_2_OH), 62.69 (CH_2_OH), 71.28 (C-5), 72.35 (C-4), 74.51 (C-1), 125.43 (C-3), 132.92 (C-2), 170.60 (C=O).

*1-C-(4-O-Acetyl-2,3,6-trideoxy-α-l-erytro-hex-2-en-pyranosyl)-5-hydroxypentane,* (**10a**): Yellow oil, (85%), 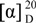 −88.73° (c 1.63, CHCl_3_), ^1^H-NMR (300 MHz, CDCl_3_) δ (ppm): 1.24 (d, 3H, *J* = 6.6 Hz, CH_3_), 1.33–1.70 (m, 8H, CH_2_CH_2_CH_2_CH_2_), 1.73 (s 1H, OH), 2.08 (s, 3H, CH_3_CO), 3.64 (t, 2H, *J* = 6.8 Hz, CH_2_OH), 3.89 (dq, 1H, *J* = 6.5 Hz, *J* = 4.7 Hz, H-5), 4.13 (m, 1H, H-1), 4.88 (m, 1H, H-4), 5.75 (ddd, 1H, *J* = 10.3 Hz, *J* = 3.5 Hz, *J* = 2.1 Hz, H-3), 5.91 (ddd, 1H, *J* = 9.4 Hz, *J* = 2.3 Hz, *J* = 1.2 Hz, H-2). ^13^C-NMR (75 MHz, CDCl_3_) δ (ppm): 16.94 (CH_3_), 21.15 (CH_3_CO), 25.48 (CH_2_CH_2_CH_2_CH_2_CH_2_OH), 25.59 (CH_2_CH_2_CH_2_CH_2_CH_2_OH), 33.56 (CH_2_CH_2_CH_2_CH_2_CH_2_OH), 33.75 (CH_2_CH_2_CH_2_CH_2_CH_2_OH), 62.67 (CH_2_OH), 68.35 (C-5), 69.71 (C-4), 70.05 (C-1), 122.48 (C-3), 134.14 (C-2), 170.74 (C=O).

*1-C-(4-O-Acetyl-2,3,6-trideoxy-β-l-erytro-hex-2-en-pyranosyl)-5-hydroxypentane* (**10b**): Yellow oil, (87%), 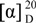 −119.32° (c 1.294, CHCl_3_), ^1^H-NMR (300 MHz, CDCl_3_) δ (ppm): 1.24 (d, 3H, *J* = 6.4 Hz, CH_3_), 1.30–1.65 (m, 8H, CH_2_CH_2_CH_2_CH_2_), 1.79 (s 1H, OH), 2.08 (s, 3H, CH_3_CO), 3,57 (dq, 1H, *J* = 8.7, *J* = 6.2Hz, H-5), 3.64 (t, 2H, *J* = 6.5 Hz, CH_2_OH), 4.14 (m, 1H, H-1), 5.04 (m, 1H, H-4), 5.68 (ddd, 1H, *J* = 10.3 Hz, *J* = 2.0 Hz, *J* = 2.0 Hz, H-2), 5.79 (ddd, 1H, *J* = 10.3 Hz, *J* = 1.5 Hz, *J* = 1.5 Hz, H-3); ^13^C-NMR (75 MHz, CDCl_3_) δ (ppm): 18.43 (CH_3_), 21.12 (CH_3_CO), 25.48 (CH_2_CH_2_CH_2_CH_2_CH_2_OH), 25.70 (CH_2_CH_2_CH_2_CH_2_CH_2_OH), 33.58 (CH_2_CH_2_CH_2_CH_2_CH_2_OH), 35.18 (CH_2_CH_2_CH_2_CH_2_CH_2_OH), 62.78 (CH_2_OH), 71.31 (C-5), 72.30 (C-4), 74.50 (C-1), 125.28 (C-3), 133.03 (C-2), 170.61 (C=O).

### 3.7. General Procedure for the Transformation of Hydroxyl Group to Bromide

The solution of triphenylphosphine (TPP, 289 mg, 1.1 mmol) in dichloromethane (5.0 mL) was stirred at room temperature and treated with bromine (54 μL, 1.05 mmol). After disappearance of the color, a solution of compound **8a** or **8b**, (230 mg, 1 mmol) in dichloromethane (4.0 mL) was added and stirring was continued for 5min. The mixture was concentrated and the residue was chromatographed on a silica gel column (hexane/acetone 20:1) to give product **11a** or **11b**.

*1-C-(4-O-Acetyl-2,3,6-trideoxy-α-l-erytro-hex-2-en-pyranosyl)-3-bromopropane* (**11a**): Yellow oil; (88%), 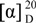 −62.20° (c 0.947, CHCl_3_); ^1^H-NMR (600 MHz, CDCl_3_) δ (ppm): 1,24 (d, *J* = 6.6 Hz, 3H, CH_3_), 1.62–2.15 (m, 4H, CH_2_CH_2_), 2.09 (s, 3H, CH_3_CO), 3.48 (m, 2H, CH_2_Br), 3.90 (dq, *J* = 6.5 Hz and *J* = 4.7 Hz, 1H, H-5), 4.18 (m, 1H, H-1), 4.89 (m, 1H, H-4), 5.79 (ddd, *J* = 10.3 Hz, 3.5 Hz, and 2.0 Hz, 1H, H-3), 5.89 (ddd, *J* = 10.3 Hz, 2.1 Hz and 1.1 Hz, 1H, H-2); ^13^C-NMR (150 MHz, CDCl_3_) δ (ppm): 16.9 (CH_3_), 21.2 (CH_3_CO), 29.0 (CH_2_CH_2_CH_2_Br), 31.9 (CH_2_CH_2_CH_2_Br), 33.8 (CH_2_Br), 68.5 (C-5), 69.4 (C-4), 69.5 (C-1), 123.1 (C-3), 134.7 (C-2), 170.7 (C=O). HRMS: calcd for [M+Na]^+^: *m/z* 315.0208, found: *m/z* 315.0205.

*1-C-(4-O-Acetyl-2,3,6-trideoxy-β-l-erytro-hex-2-en-pyranosyl)-3-bromopropane* (**11b**): Yellow oil, (92%); 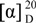 −112.58° (c 1.27, CHCl_3_); ^1^H-NMR (600 MHz, CDCl_3_) δ (ppm): 1.23 (d, *J* = 6.3 Hz, 3H, CH_3_), 1.54–1.81 (m, 2H, CH_2_CH_2_CH_2_Br), 1.91–2.05 (m, 2H, CH_2_CH_2_CH_2_Br), 2.08 (s, 3H, CH_3_CO), 3.44 (t, 2H, *J* = 6.7 Hz, CH_2_Br), 3.56 (dq, 1H *J* = 8.7 Hz and 6.2 Hz, H-5), 4.18 (m, 1H, H-1), 5.03 (m, 1H, H-4), 5.71 (ddd, *J* = 10.4 Hz, 1.7 Hz, and 1.7 Hz, 1H, H-2), 5.77 (ddd, *J* = 10.3 Hz, 1.2 Hz, and 1.2 Hz, 1H, H-3); ^13^C-NMR (150 MHz, CDCl_3_) δ (ppm): 18.4 (CH_3_), 21.1 (CH_3_CO), 28.1 (CH_2_CH_2_CH_2_Br), 33.6 (CH_2_CH_2_CH_2_Br), 33.9 (CH_2_Br), 71.1 (C-5), 72.34(C-4), 74.0 (C-1), 126.0 (C-3), 132.5 (C-2), 170.5 (C=O). HRMS: calcd for [M+Na]+: *m/z* 315.0208, found: *m/z* 315.0204.

*1-C-(4-O-Acetyl-2,3,6-trideoxy-α-l-erytro-hex-2-en-pyranosyl)-4-bromobutane* (**12a**): Yellow oil, (91%); 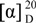 −52.20° (c 1.63, CHCl_3_), ^1^H-NMR (600 MHz, CDCl_3_) δ (ppm): 1.24 (d, 3H, *J* = 6.3Hz, CH_3_), 1.47–1.73 (m, 4H, CH_2_CH_2_), 1.83–1.98 (tt, 2H, *J* = 7.0 Hz, CH_2_CH_2_Br) 2.08 (s, 3H, CH_3_CO), 3.42 (t, 2H, *J* = 6,8 Hz, CH_2_Br), 3.89 (m, 1H, H-5), 4.14 (m, 1H, H-1), 4.88 (m, 1H, H-4), 5.77 (ddd, 1H, *J* = 10.3 Hz, *J* = 3.7 Hz, *J* = 2.2Hz, H-3), 5.90 (m, 1H, H-2); ^13^C-NMR (150 MHz, CDCl_3_) δ (ppm): 16.91 (CH_3_), 21.19 (CH_3_CO), 24.40 (CH_2_CH_2_CH_2_CH_2_Br), 32.57 (CH_2_CH_2_CH_2_CH_2_Br), 32.76 (CH_2_Br), 33.52 (CH_2_CH_2_CH_2_CH_2_Br), 68.52 (C-5), 69.58 (C-4), 69.77 (C-1), 122.77 (C-3), 133.90 (C-2), 170.69 (C=O).

*1-C-(4-O-Acetyl-2,3,6-trideoxy-β-l-erytro-hex-2-en-pyranosyl)-4-bromobutane* (**12b**): Yellow oil, (90%); 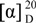 −84.67° (c 1.807, CHCl_3_); ^1^H-NMR (600 MHz, CDCl_3_) δ (ppm): 1.23 (d, 3H, *J* = 6.3 Hz, CH_3_), 1.49–1.62 (m, 4H, CH_2_CH_2_), 1.82–1.96 (m, 2H, CH_2_CH_2_Br), 2.08 (s, 3H, CH_3_CO), 3.41 (t, 2H, *J* = 6.8 Hz, CH_2_Br), 3.56 (dq, 1H, *J* = 8.8 *J* = 6.1 Hz, H-5), 4.14(m, 1H, H-1), 5.03 (m, 1H, H-4), 5.69 (ddd, 1H, *J* = 10.3 Hz, *J* = 2.0 Hz, *J* = 2.0Hz, H-2), 5.77 (ddd, 1H, *J* = 10.6 Hz, *J* = 1.8 Hz, *J* = 1.8Hz, H-3). ^13^C-NMR (150 MHz, CDCl_3_) δ (ppm): 18.45(CH_3_), 21.11(CH_3_CO), 23.45 (CH_2_CH_2_CH_2_CH_2_Br), 32.72 (CH_2_Br), 33.56 (CH_2_CH_2_CH_2_CH_2_Br), 34.30 (CH_2_CH_2_CH_2_CH_2_Br), 71.22 (C-5), 72.33 (C-4), 74.26 (C-1) 125.65 (C-3), 132.73 (C-2), 170.51 (C=O).

*1-C-(4-O-Acetyl-2,3,6-trideoxy-α-l-erytro-hex-2-en-pyranosyl)-5-bromopentane* (**13a**): Yellow oil, (91%); 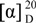 −100.93° (c 0.86, CHCl_3_); ^1^H-NMR (600 MHz, CDCl_3_) δ (ppm): 1.24 (d, 3H, *J* = 6.6 Hz, CH_3_), 1.36–1.70 (m, 6H, CH_2_CH_2_CH_2_), 1.88 (q, 2H, *J* = 7.0 Hz, CH_2_CH_2_Br) 2.08 (s, 3H, CH_3_CO), 4.02 (t, 2H, *J* = 6.7 Hz, CH_2_Br), 3.91 (dq, 1H, *J* = 6.5 Hz, *J* = 4.7 Hz, H-5), 4.13 (m, 1H, H-1), 4.88 (m, 1H, H-4), 5.77 (ddd, 1H, *J* = 10.6 Hz, *J* = 3.5 Hz, *J* = 1.7Hz, H-3), 5.94 (ddd, 1H, *J* = 10.6 Hz, *J* = 2.3 Hz, *J* = 1.2 Hz, H-2). ^13^C-NMR (150 MHz, CDCl_3_) δ (ppm): 16.94 (CH_3_), 21.19 (CH_3_CO),24.91 (CH_2_CH_2_CH_2_CH_2_CH_2_Br), 28.88 (CH_2_CH_2_CH_2_CH_2_CH_2_Br), 32.63 (CH_2_CH_2_CH_2_CH_2_CH_2_Br), 33.45 (CH_2_CH_2_CH_2_CH_2_CH_2_Br), 33.74 (CH_2_Br), 68.42 (C-5), 69.63 (C-4), 69.90 (C-1),122.58 (C-3), 134.07 (C-2), 170.69 (C=O).

*1-C-(4-O-Acetyl-2,3,6-trideoxy-β-l-erytro-hex-2-en-pyranosyl)-3-bromopentane* (**13b**): Yellow oil, (94%); 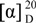 −126.07° (c 1.417, CHCl_3_), ^1^H-NMR (300 MHz, CDCl_3_) δ (ppm): 1.24 (d, 3H, *J* = 6.4 Hz, CH_3_), 1.35–1.62 (m, 6H, CH_2_CH_2_CH_2_), 1.80–1.93 (m, 2H, CH_2_CH_2_Br) 2.08 (s, 3H, CH_3_CO), 3.41 (t, 2H, *J* = 6.8 Hz, CH_2_Br), 3,57 (dq, 1H, *J* = 8.6 Hz, *J* = 6.2 Hz, H-5), 4.14 (m, 1H, H-1), 5.04 (m, 1H, H-4), 5.69 (ddd, 1H, *J* = 10.3 Hz, *J* = 2.0 Hz, *J* = 2.0 Hz, H-2), 5.78 (ddd, 1H, *J* = 10.3 Hz, *J* = 1.5 Hz, *J* = 1.5Hz, H-3); ^13^C-NMR (75 MHz, CDCl_3_) δ (ppm): 18.46 (CH_3_), 21.14 (CH_3_CO), 23.87 (CH_2_CH_2_CH_2_CH_2_CH_2_Br), 28.10 (CH_2_CH_2_CH_2_CH_2_CH_2_Br), 32.65 (CH_2_CH_2_CH_2_CH_2_CH_2_Br), 33.83 (CH_2_Br), 35.03 (CH_2_CH_2_CH_2_CH_2_CH_2_Br), 71.27 (C-5), 72.32 (C-4), 74.40 (C-1), 125.46 (C-3), 132.94 (C-2), 170.56 (C=O).

### 3.8. General Procedure for Synthesis of 2,3-Enopyranosyl C-Linked Conjugates of Genistein

A solution of C-glycoside **11**–**13** (1 mmol), tetra *n*-butylammonium salt of genistein **14** (1,2 mmol, 614 mg) in DMF (4 mL) was stirred at temperature 45–50 °C to a complete dissolution of salt. DMF was evaporated under reduced pressure and the residue was dissolved in CHCl_3,_ washed with water. Organic layer was dried over Na_2_SO_4_, evaporated on rotary evaporator. The crude product was purified by silica gel column chromatography (gradient of CH_2_Cl_2_/acetone 30:1→10:1) to give compound **15**–**17**.

*5-Hydroxy-7-O-[1-C-(3-(4-O-acetyl-2,3,6-trideoxy-α-l-erythro-heks-2-en-pyranosyl) propyl]–3–(4'–hydroxyphenyl) chromen–4–on* (**15a**): Solid, m.p. 126–129 °C, (77%); 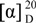 −35.82° (c 0.67, CHCl_3_), HRMS: calcd for [M+Na]^+^: 489.5, found: *m/z* = 489.0. ^1^H-NMR (600 MHz, CDCl_3_) δ (ppm): 1.27 (d, 3H, *J* = 6.6 Hz, CH_3_), 1.68–2.05 (m, 4H, CH_2_CH_2_), 2.10 (s, 3H, CH_3_CO), 3.91 (dq, 1H, *J* = 6.6, *J* = 4.6 Hz, H-5), 4.06 (t, 2H, *J* = 6.1 Hz, OCH_2_), 4.24 (m, 1H, H-1), 4.91 (m, 1H, H-4), 5.81 (ddd, 1H, *J* = 10.3 Hz, *J* = 3.6 Hz, *J* = 2.0 Hz, H-3), 5.94 (ddd, 1H, *J* = 10.3 Hz, *J* = 2.1 Hz, *J* = 1.1 Hz, H-2), 6.29 (s, 1H, 4'-OH), 6,36 (d, 1H, *J* = 2.4 Hz,, H-8), 6,38 (d, 1H, *J* = 2.4 Hz, H-6), 6.84 (AA'XX', 2H, *J* = 8.7 Hz, 2H, H-3'g, H-5'g), 7.34 (AA'XX', 2H, *J* = 8.7 Hz,, 2H, H-2'g, H-6'g), 7.84 (s, 1H, H-2g), 12.80 (s, 1H, 5g-OH); ^13^C-NMR (150 MHz, CDCl_3_) δ (ppm): 16.82 (CH_3_), 21.19 (CH_3_CO), 25.23 (CH_2_CH_2_CH_2_O), 29,91 (CH_2_CH_2_CH_2_O), 68.24 (C-5), 68.72 (CH_2_O), 69.53 (C-4), 69.55 (C-1), 92.82 (C-8g), 98.64 (C-6g), 106.12 (C-4a), 115.67 (C-3'g, C-5'g), 122.52 (C-1'g), 122.85 (C-3), 123.71 (C-3g), 130.22 (C-2'g, C-6'g), 133.82 (C-2), 152.80 (C-2g), 156.26 (C-4'g), 157.94 (C-8a), 162.50 (C-5g), 164.99 (C-7g) 171.00 (C=O), 180.94 (C-4g).

*5-Hydroxy-7-O-[3-(1-C-4-O-acetyl-2,3,6-trideoxy-α-l-erytro-heks-2-en-pyranosyl)propyl]-3-(4-hydroxyphenyl)chromen-4-on* (**15b**): Solid, m.p. 166–168 °C, (75%); 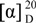 −74.69° (c 0.64, CHCl_3_) HRMS: calcd for [M+Na]^+^: 489.5, found: *m/z* = 489.0, ^1^H-NMR (600 MHz, CDCl_3_) δ (ppm): 1.26 (d, 3H, **J** = 6.2 Hz, CH_3_), 1.59-2.00 (m, 4H, CH_2_CH_2_) 2.10 (s, 3H, CH_3_CO), 3.61 (dq, 1H, *J* = 8.6, *J* = 6.2 Hz, H-5), 4.07 (t, 2H, *J* = 6.3 Hz, CH_2_O), 4.25 (m, 1H, H-1), 5.07 (m, 1H, H-4), 5.54 (s, 1H, 4'-OH), 5.73 (ddd, 1H *J* = 10.3 Hz *J* = 1.8 Hz, H-2), 5.81 (ddd, 1H, *J* = 10.3 Hz, *J* = 1.4 Hz, *J* = 1.4 Hz, H-3), 6.36 (d, 1H, *J* = 2.2 Hz, H-8), 6.39 (d, 1H, *J* = 2.2 Hz, H-6), 6.87 (AA'XX', 2H, *J* = 8.6 Hz, 2H, H-3'g, H-5'g), 7.37 (AA'XX', 2H, *J*=8.6 Hz, 2H, H-2'g, H-6'g), 7.85(s, 1H, H-2g), 12.80(s, 1H, 5g-OH); ^13^C-NMR (150 MHz, CDCl_3_) δ (ppm): 18.50 (CH_3_), 21.17 (CH_3_CO), 24.32 (CH_2_CH_2_CH_2_O), 31.49 (CH_2_CH_2_CH_2_O), 68.48 (CH_2_O), 71.26 (C-5), 72.43 (C-4), 74.08 (C-1), 92.87 (C-8g), 98.68 (C-6g), 106.19 (C-4a), 115.65 (C-3'g, C-5'g), 122.94 (C-1'g), 123.69 (C-3g) 125.99 (C-3), 130.33 (C-2'g, C-6'g), 132.65 (C-2), 152.77 (C-2g), 156.04 (C-4'g), 157.99 (C-8a), 162.33 (C-5g), 165.06 (C-7g), 170.74 (C=O), 180.90 (C-4g).

*5-Hydroxy-7-O-[4-(1-C-4-O-acetyl-2,3,6-trideoxy-α-l-erythro-heks-2-en-pyranosyl)butyl]-3-(4'-hydroxyphenyl)chromen-4-on* (**16a**): Yellow oil, (70%), 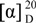 −37.51°(c 1.27, CHCl_3_), HRMS: calcd for [M+Na]^+^: 503.5, found: *m/z* = 503.1; ^1^H-NMR (600 MHz, CDCl_3_) δ (ppm): 1.27 (d, 3H, *J* = 6.6 Hz, CH_3_), 1.5–2.00 (m, 6H, CH_2_CH_2_CH_2_), 2.09 (s, 3H, CO-CH_3_), 3.95 (dq, 1H, *J* = 6.6 Hz, *J* = 4.4 Hz, H-5), 4.01 (t, 2H, *J* = 6.1 Hz, OCH_2_), 4.19 (m, 1H, H-1), 4.90 (m, 1H, H-4), 5.79 (ddd, 1H, *J* = 10.3 Hz, *J* = 3.6 Hz, *J* = 2.0 Hz, H-3), 5.94 (ddd, 1H, *J* = 10.3 Hz, *J* = 2.0 Hz, *J* = 1.1Hz, H-2), 6.35 (d, 1H, *J* = 2.2 Hz, H-8), 6.37 (d, 1H, *J* = 2.2Hz, H-6), 6.71 (s, 1H, 4'-OH), 6.87 (AA'XX', 2H, *J* = 8.6 Hz, 2H, H-3'g, H-5'g), 7.35 (AA'XX', 2H, *J* = 8.4 Hz, 2H, H-2'g, H-6'g), 7.83 (s, 1H, H-2g), 12.82 (s, 1H, 5g-OH), ^13^C-NMR (150 MHz, CDCl_3_) δ (ppm): 16.84 (CH_3_), 21.21 (CH_3_CO), 22.24 (CH_2_CH_2_CH_2_CH_2_O), 28.77 (CH_2_CH_2_CH_2_CH_2_O), 33.32 (CH_2_CH_2_CH_2_CH_2_O), 68.40 (C-5), 68.70 (CH_2_O), 69.62 (C-4), 69.80 (C-1), 92.79 (C-8g), 98.58 (C-6g), 106.09 (C-4a), 115.65 (C-3'g, C-5'g), 122.41(C-1'g) 122.58 (C-3), 123.68 (C-3g), 130.18 (C-2'g, C-6'g) 134,02 (C-2), 152.71 (C-2g), 156.47 (C-4’g), 157.91 (C-8a), 162.52 (C-5g), 164.99 (C-7g) 170.99 (C=O), 180.90 (C-4g).

*5-Hydroxy-7-O-[4-(1-C-4-O-acetyl-2,3,6-trideoxy-α-l-erytro-hex-2-en-pyranosyl)butyl]-3-(4'-hydroxyphenyl)chromen-4-on* (**16b**): Yellow oil (77%); 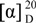 −47.20° (c 1.017, CHCl_3_), HRMS: [M+Na]^+^: *m/z* = 503.1; ^1^H-NMR (600 MHz, CDCl_3_) δ (ppm): 1.25 (d, 3H, *J* = 6.2 Hz, CH_3_), 1.52–1.66 (m, 4H, CH_2_CH_2_), 1.77–1.88 (m, 2H, CH_2_CH_2_O), 2.09 (s, 3H, CH_3_CO), 3.59 (dq, ^1^H, *J* = 8.8 Hz, *J* = 6.2 Hz, *J* = 6.2 Hz, H-5), 4.01(t, 2H, *J* = 6.5 Hz, *J* = 6.2 Hz, OCH_2_), 4.18 (m, ^1^H, H-1), 5.06 (m, ^1^H, H-4), 5.71 (ddd, ^1^H, *J* = 10.3 Hz, *J* = 2.1 Hz, *J* = 2.1 Hz, *J* = 6.2 Hz, H-2), 5.79 (ddd, *J* = 10.3 Hz, *J* = 1.6 Hz, *J* = 1.6 Hz, *J* = 6.2 Hz, H-3), 6,35 (d, ^1^H, *J* = 2.1 Hz, *J* = 6.2 Hz, H-8), 6.37 (d, ^1^H, *J* = 2.1 Hz, *J* = 6.2 Hz, H-6), 6.02 (s, ^1^H, 4'-OH), 6.85 (AA'XX', 2H, *J* = 9.0 Hz, 2H, H-3'g, H-5'g), 7.35 (AA'XX', 2H, *J* = 8.4 Hz, 2H, H-2'g, H-6'g), 7.83 (s, ^1^H, H-2g), 12.79 (s, ^1^H, 5g-OH); ^13^C-NMR (150 MHz, CDCl_3_) δ (ppm): 18.48 (CH_3_), 21.18 (CH_2_CH_2_CH_2_CH_2_O), 21.29 (CH_3_CO), 28.92 (CH_2_CH_2_CH_2_CH_2_O), 34.87 (CH_2_CH_2_CH_2_CH_2_O), 68.50 (CH_2_O), 71.38 (C-5), 72.44 (C-4), 74.48 (C-1), 92.88 (C-8g), 98.70 (C-6g), 106.15 (C-4a), 115.71 (C-3'g, C-5'g), 122.70 (C-1'g), 122.73 (C-3g), 125.62 (C-3), 130.29 (C-2'g, C-6'g), 132,86 (C-2), 152.82 (C-2g), 156.82 (C-4'g), 157.99 (C-8a), 162.56 (C-5g), 165.11 (C-7g), 170.85 (C=O), 180.9 (C-4g).

*5-Hydroxy-7-O-[5-(1-C-4-O-acetyl-2,3,6-trideoxy-α-l-erythro-hex-2-en-pyranosyl)pentyl]-3-(4'-hydroxyphenyl)chromen-4-on* (**17a**): Solid, m.p. 116–125 °C, (69%); 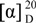 −39.09° (c 0.66, CHCl_3_), HRMS: calcd for [M+Na]^+^: 517.2 found: *m/z* = 517.5; ^1^H-NMR (600 MHz, CDCl_3_) δ (ppm): 1.25 (d, 3H, *J* = 6.6 Hz, CH_3_), 1.44–1.72 (m, 6H, CH_2_CH_2_CH_2_), 1.83 (q, 2H, *J* = 6.8 Hz, CH_2_CH_2_O), 2.09 (s, 3H, CH_3_CO), 3.91 (dq, ^1^H, *J* = 6.6 *J* = 4.8 Hz, H-5), 4.02 (t, 2H, *J* = 6.1 Hz, OCH_2_), 4.15 (m, ^1^H, H-1), 4.90 (m, ^1^H, H-4), 5.77 (ddd, ^1^H *J* = 10.4 Hz, *J* = 3.5 Hz, *J* = 2.1 Hz, H-3), 5.91 (ddd, ^1^H, *J* = 10.3 Hz, *J* = 2.3 Hz, *J* = 1.3 Hz, H-2), 6.36 (d, ^1^H, *J* = 2.2 Hz, H-8), 6.38 (d, ^1^H, *J* = 2.2 Hz, H-6), 6.90 (AA'XX', 2H, *J* = 8.4 Hz, H-3'g, H-5'g), 7.41 (AA'XX', 2H, *J* = 8.4 Hz, H-2'g, H-6'g), 7.85 (s, ^1^H, H-2g), 12.81 (s, ^1^H, 5g-OH); ^13^C-NMR (150 MHz, CDCl_3_) δ (ppm): 16.92 (CH_3_), 21.28 (CH_3_CO), 25.43 (CH_2_CH_2_CH_2_CH_2_CH_2_O), 25.86 (CH_2_CH_2_CH_2_CH_2_CH_2_O), 28.84 (CH_2_CH_2_CH_2_CH_2_CH_2_O), 33.59 (CH_2_CH_2_CH_2_CH_2_CH_2_O), 68.50 (C-5), 68.66 (CH_2_O), 69.74 (C-4), 69.99 (C-1), 92.85 (C-8g), 98.64 (C-6g), 106.07 (C-4a), 115.71 (C-3'g, C-5'g), 122.46 (C-1'g, C-3), 123.70 (C-3g), 130.24 (C-2'g, C-6'g) 134.21 (C-2), 152.83 (C-2g), 156.40 (C-4'g), 157.95 (C-8a), 162.46 (C-5g), 165.09 (C-7g), 171.12 (C=O), 180.96 (C-4g).

*5-Hydroxy-7-O-[5-(1-C-4-O-acetyl-2,3,6-trideoxy-α-l-erytro-hex-2-en-pyranosyl)pentyl]-3-(4-hydroxyphenyl)chromen-4-on* (**17b**): Solid, m.p. 152–154° C, (71%), 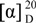 −65.45° (c 0.64, CHCl_3_), HRMS: calcd for [M+Na]^+^: 517.5 found: 517.2; ^1^H-NMR (600 MHz, CDCl_3_) δ (ppm): 1.25 (d, 3H, *J* = 6.2 Hz, CH_3_), 1.42–1.62 (m, 6H, CH_2_CH_2_CH_2_), 1.78–1.85(m, 2H, CH_2_CH_2_O), 2.09 (s, 3H, CH_3_CO), 3.57 (dq, ^1^H, *J* = 8.7 Hz, *J* = 6.2 Hz, H-5), 4.01 (t, 2H, *J* = 6.5 OCH_2_), 4.16 (m, ^1^H, H-1), 5.05 (m, ^1^H, H-4), 5.67 (s, ^1^H, 4'-OH), 5.70 (ddd, ^1^H, *J* = 10.4 Hz, *J* = 2.1 Hz, H-2), 5.79 (ddd, *J* = 10.3 Hz, *J* = 1.6 Hz, H-3), 6.36 (d, ^1^H, *J* = 2.2 Hz, H-8), 6,38 (d, ^1^H, *J* = 2.2 Hz, H-6), 6.85 (AA'XX', 2H, H-3'g, H-5'g *J* = 8.8 Hz), 7.36 (d, 2H, H-2'g, H-6'g *J* = 8.7 Hz), 7.84 (s, ^1^H, H-2g), 12.79 (s, ^1^H, 5g-OH); ^13^C-NMR (150 MHz, CDCl_3_) δ (ppm): 18.49 (CH_3_ram), 21.18 (CH_3_CO), 24.43 (CH_2_CH_2_CH_2_CH_2_CH_2_O), 25.96 (CH_2_CH_2_CH_2_CH_2_CH_2_O), 28.85 (CH_2_CH_2_CH_2_CH_2_CH_2_O), 35.15 (CH_2_CH_2_CH_2_CH_2_CH_2_O), 68.44 (CH_2_O), 71.41 (C-5), 72.41 (C-4), 74.55 (C-1), 92.90 (C-8g), 98.68 (C-6g), 106.13 (C-4a), 115.68 (C-3’g, C-5’g), 122.83 (C-1’g), 123.71 (C-3g), 125.45 (C-3), 130.31 (C-2'g, C-6'g), 133,02 (C-2), 152.79 (C-2g), 156.11 (C-4'g), 158.00 (C-8a), 162.59 (C-5g), 165.16 (C-7g), 170.80 (C=O), 180.94 (C-4g).

### 3.9. Anticancer Activity in Vitro

#### 3.9.1. Cell Viability

Cell viability was estimated using an MTT (3-[4,5-dimethylthiazol-2-y]2,5-diphenyltetrazolium bromide) assay (Sigma–Aldrich, Hamburg, Germany), according to the supplier’s protocol. Shortly, 24 h before addition of the tested compounds, the cells were plated in 96-well plates (Sarstedt, Nümbrecht, Germany) at the density of 2 × 10^3^ cells per well. Assays were performed after 72 h of continuous exposure to varying concentrations of the tested agents. Each compound in each concentration was tested in hexaplicate in a single experiment, which was repeated at least three times. Viability of cells was expressed as a percentage versus vehicle control (DMSO treatment). IC_50_ was defined as a concentration of a drug that decreased cell viability by 50%. 

#### 3.9.2. Cell Cycle Analysis

Cells in subconfluent proliferating cultures were incubated with the tested compounds continuously for 24 h. Floating cells were collected and added to adherent cells harvested by trypsinization. Cells were washed with PBS and then fixed in ice-cold ethanol (70%) for 30 min, treated with RNase (100 μg/mL) and stained with propidium iodide (PI) (100 μg/mL). DNA content was analyzed using Becton Dickinson FACSCanto cytometer (BD Company, Franklin Lakes, NJ, USA) to monitor the cell cycle changes. Experiments were repeated at least three times.

#### 3.9.3. Microscope Analysis

Cells, collected as described above, were cytospinned, fixed with cold methanol, stained with solution of DAPI (3 μM). Slides were mounted in DAKO^®^Fluorescent Mounting Medium (Dako, Carpinteria, CA, USA) and examined under an ECLIPSE E800 Nikon microscope (Nikon, Tokyo, Japan) using an objective 40×. Mitotic cells and cells with fragmented nuclei (at least one large micronucleus present in the cell) were counted among 1000 cells of the specimen. The *in vitro* micronucleus assay with size-classified micronucleus counting was performed according to the method described elsewhere [47]. The experiments were repeated three times. 

#### 3.9.4. Statistics

Three separate experiments were performed in six replicates for each sample concentration to assess cytotoxicity. Curve fitting was performed using nonlinear regression model in GraphPad Prism software. Data were expressed as means ± S.D. Three separate experiments were performed for cell cycle analysis. Data were expressed as means ± S.D. Three independent experiments were performed for microscopy examination of nuclei morphology. Data were expressed as means ± S.D. Statistical comparison between untreated cells and cells treated with the tested compounds was performed using one-way ANOVA (GraphPad Prism software, GraphPad Software, Inc., La Jolla, CA USA).

## 4. Conclusions

We have elaborated simple and effective method of synthesis for intermediate alkyl aryl organozinc iodides and their coupling with 3,4-di-*O*-acetyl-l-rhamnal, affording 2,3-unsaturated C-glycosides in good yield, and rather insignificant stereoselectivity. Preparative separation of isomers can be easily achieved by classical silica gel chromatography. These novel synthetic derivatives of the parent isoflavone exhibit cytotoxicity towards selected cell lines, which varies with such structural parameters as anomeric configuration and carbon spacer length. The new derivatives also significantly alter cell cycle and cause mitotic perturbations not observed for genistein itself. It seems that remarkable changes in activity of unsaturated genistein glycosides, when compared to the parent isoflavone, are largely retained after the switch from *O*- to C-conjugation.
